# Extraction of Child by a Novel Process

**Published:** 1851-09

**Authors:** A. E. Ames


### ARTICLE V.

### *Extraction of Child by a Novel Process.*

[Dr. Samuel Thompson sends us the following:]

I reply to an application for facts on Obstetrics for the Committee of the American Medical Association, I received from Dr. A. E. Ames, of Roscoe, the following. It came too late for its intended object—it is at your service:

"Mrs. H., in labor with 10th child; in labor 7 hours; pains very hard, progress slow. First presentation of Baudeloque; previous to labor, the labia majora and minora had become some swollen, and as labor progressed the swelling increased, in consequence of the enlargement of the parts.

The child's head being very large, completely filling up the pelvic region, and there being no prospect of a natural termination of the labor, and it being impossible to apply the forceps, I determined to perform craniotomy. After having made an incision through the scalp 2½ inches in length, I raised the scalp and passed two fingers of my right hand under it far enough that when I made extension the force would not come against the edges of the incision; then placing my left hand against the perinæum, I made extension with my right. This had a tendency to elongate the head of the child, and aided by the pains, which were very good from the first, the child was born alive. The wound was dressed with simple dressings.

Thus may a child, in my opinion, be saved. This may not be a *new act* in Obstetrics; if it is, please place the same before the public eye. A. E. AMES.

*March* 20*th*, 1851.

